# Paying for the Greater Good?—What Information Matters for Beijing Consumers’ Willingness to Pay for Plant-Based Meat?

**DOI:** 10.3390/foods11162460

**Published:** 2022-08-15

**Authors:** Hongsha Wang, Qihui Chen, Chen Zhu, Jiale Bao

**Affiliations:** 1 College of Economics and Management, China Agricultural University, Beijing 100083, China; 2 Beijing Food Safety Policy & Strategy Research Base, China Agricultural University, Beijing 100083, China

**Keywords:** information shock, sustainable food consumption, plant-based meat, food attributes, willingness to pay, choice experiment

## Abstract

Promoting the transition from animal meat to plant-based food consumption has significant benefits for public health and environmental sustainability. This study, involving 526 consumers from Beijing, China, explores how food attributes and information may affect consumers’ food choices concerning plant-based meat products. A discrete choice experiment was conducted using burgers with five attributes (meat patties, flavor, sodium content, energy, and price) as the focal product. Separate messages on nutrition, food safety, and the environmental issues related to plant-based meat consumption were also randomly provided to consumers to help examine the role of information. Our findings suggest that Beijing consumers’ awareness of plant-based meat is relatively low at present, and they show a negative preference toward plant-based meat consumption relative to that of conventional meat. However, consumers’ willingness to pay for plant-based meat significantly increased after nutrition information was provided, but it was not responsive to the provision of food safety or environmental information. These findings suggest that to promote plant-based meat consumption, information closely related to consumers’ personal interests rather the “greater good” should be provided, at least in the context of Beijing, China.

## 1. Introduction

No one lives without food. Yet food consumption, especially the consumption of animal products, creates various externalities that negatively impact the environment. At present, food consumption is responsible for nearly 30% of global energy consumption, and food-related greenhouse gas emissions account for about 22% of total global emissions [1]. As increasingly more countries have realized the pressing need to address the environmental threats, food safety concerns, and health risks associated with animal-product consumption [2,3,4,5], how to cope with these challenges while maintaining the quality of food consumption has become a hotly debated topic.

The livestock sector attracts considerable attention. First of all, it is one of the major contributors to greenhouse gas emissions. According to different estimates [6,7,8], livestock production was responsible for 5.8–18% of total global emissions in the last decade. Meanwhile, excessive (red and processed) meat consumption imposes threats on consumer health, potentially raising the risks of chronic illnesses such as type-2 diabetes mellitus, coronary heart disease, stroke, and colorectal cancer among food consumers [5,9]. These problems have led many countries (e.g., the United States and Germany) and international organizations (e.g., the Food and Agriculture Organization of the United Nations and the Nordic Ministerial Conference) to provide guidelines for reducing meat consumption [10,11,12,13,14].

One popular proposal is to promote the consumption of meat substitutes, especially plant-based foods, including plant-based meat products [14,15,16]. Usually consumed in the form of burger patties, crumbles, and sausages, plant-based meat is made with plant-based proteins extracted from peas, wheat, and soybeans, with ingredients and additives (e.g., flavoring and colorants) that mimic the flavor, taste, and appearance of conventional meat [17]. Compared with beef and other red meat products, the consumption of plant-based meat has a clear environmental advantage. For example, global greenhouse gas emissions from plant-based foods are only half those from animal-based foods [18]. In addition, it takes only about one-twentieth of the land resources needed to produce one unit of conventional meat protein to produce one unit of plant-based protein [19].

However, whether plant-based meat can be commercially successful hinges on consumers’ reactions to this novel product. Many studies have been conducted to evaluate consumer acceptance of plant-based meat alternatives in developed countries. For example, Neff et al. [20] found that consumers in the United States are willing to consume less meat and more meat alternatives. Another study done in the United States, by Van Loo et al. [21], showed that consumers tend to have stronger preferences for plant-based meat alternatives than for lab-grown alternatives. Although meat-eating consumers in Germany, France, and the United Kingdom have negative attitudes toward vegetarian and vegan lifestyles in general, they rated higher for the nutritional and environmental advantages of peas and algae over beef [22]. In Belgium, the proportion of consumers who said existing plant-based meat alternatives met their needs increased significantly from 44% in 2019 to 51% in 2020 [23]. Insightful as existing studies are, little attention has been paid to developing countries, including China, where the food markets may well be different than those in developed countries. Although plant-based meat products have entered China’s food market recently, the market for plant-based meat is not yet mature. As such, Chinese consumers’ preferences regarding plant-based meat products remain a largely unexplored question.

Generally speaking, consumer preferences for meat alternatives can be driven by various factors. Gender is one such factor. Compared with male consumers, female consumers tend to have a stronger preference for plant-based food [24]. When it comes to meat substitutes, females prefer plant-based meat substitutes, while males prefer cell-cultured meat [23]. Consumers’ beliefs also play a role. Regular meat-eaters are less likely to consume plant-based meat due to their reluctance to reduce animal protein intake; many of them do not even believe that the livestock industry is a significant contributor to climate change [25]. In contrast, consumers who believe that the overconsumption of meat is harmful are more likely to choose meat substitutes from plant sources such as beans [26]. Other factors, such as dietary motivation and consumption habits, also affect consumers’ acceptance of and willingness to consume meat substitutes [27,28,29]. While heterogeneity in consumers’ preferences for plant-based meats is relatively well-documented in the literature, the means of changing their behavior merits more investigation. Information plays a crucial role in changing consumer choices. It has been found in various settings that the provision of detailed product information can significantly change consumers’ acceptance of foods produced using new technologies [30,31,32,33], which may, in turn, change their food choice behavior. In contrast, limited information on the benefits and risks associated with the consumption of novel products may dampen consumers’ willingness to consume them. Yet the role of information in China’s nascent plant-based meat market remains to be explored.

To fill this gap, the present study investigates consumers’ preferences for plant-based meat products (burgers) in Beijing based on a Discrete Choice Experiment (DCE) involving 526 consumers, with particular attention paid to the role of information. Beijing was chosen as the study area for a number of reasons. First, as the capital city of China and an international metropolis, Beijing has both informational and cultural advantages in developing novel food markets. In July 2021, the State Council of the People’s Republic of China officially approved the construction of an international consumption center in Beijing, which has a powerful role in leading and driving consumption in China [34]. Additionally, a survey conducted by the Beijing Municipal Bureau of Ecology and Environment showed that the public’s awareness of environmental protection and environmentally friendly behaviors have been increasing in recent years in Beijing [35]. As such, a good foundation has been laid for novel and sustainable food consumption in Beijing. Research on plant-based meat consumption in Beijing can thus provide valuable references for future studies on sustainable food consumption in and outside China.

Our study contributes to the literature in three ways. First, to the best of our knowledge, this is among the few studies that examine Chinese consumers’ perceptions of and preferences for plant-based meat. Second, using plant-based meat as a case study, we provide new evidence that complements previous studies on the role of information in driving consumers’ preferences. Finally, against the backdrop of increasing public recognition of sustainable consumption with low consumer awareness and acceptance of plant-based meat, our findings may guide both practitioners and policy-makers to take informed actions, at least in the context of Beijing.

The remainder of this paper is structured as follows. The next section describes the materials and methods. Section 3 presents the results of our choice experiment. Section 4 discusses the results. The final section concludes the paper.

## 2. Materials and Methods

### 2.1. Experimental Design

#### 2.1.1. Basic Design

Since currently plant-based meat products are mostly consumed in the form of burgers in Beijing, burgers were used as the focal product in our choice experiment. Our choice experiment adopted a labeled design; all choices include four food alternatives at various prices plus a “none of these” option. The burgers to be chosen by the participants are characterized by five attributes: meat patty, flavor, sodium content, energy, and price:

*Meat patty*. For the main purpose of the study, this attribute includes two varieties: plant-based meat patty (100 g) and animal meat patty (100 g).

*Flavor*. Flavor is a key contributor to consumers’ acceptability of meat products [36]. We chose beef, pork, and chicken flavor as the levels of this attribute.

*Sodium content*. Plant-based meat patties may have higher sodium content due to the need to add salt and other seasonings to mimic the flavor of conventional meat [37,38]. To capture the effect of sodium content, we set four levels, 100 mg, 250 mg, 400 mg, and 550 mg, for this attribute.

*Energy*. It has been shown that vegetarian burgers are perceived as containing less energy than their non-vegetarian counterparts [39], and consumers might make their food choices based on calorie information [40]. Four levels were chosen to capture the effect of energy: 250 kcal, 350 kcal, 450 kcal, and 550 kcal.

*Price*. Since a single-patty meat burger in China is usually priced within the range of RMB 15–30 (RMB 1 ≈ USD 0.16 in 2021), we selected four levels of price for a burger around that range: RMB 10, RMB 20, RMB 30, and RMB 40.

**Figure 1:**
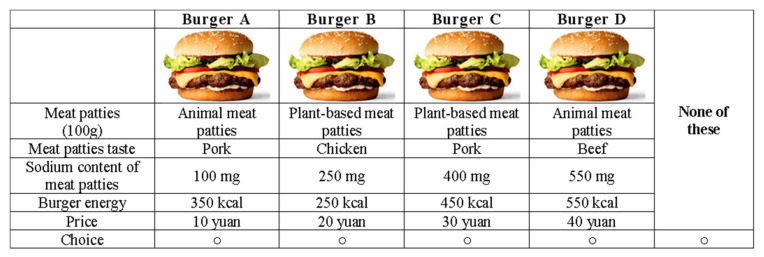
Example of a choice set.

The above design yields a total of 384 (= 2 × 3 × 4^3^) potential burger profiles. An orthogonal fractional factorial design [41] was then adopted to reduce the number of choice tasks to 32, which were then further reduced to 8 per respondent using blocking techniques (with four blocks). Participants were randomly assigned to one of the four blocks and completed eight choice tasks (in randomized order). Figure 1 illustrates an actual choice set used in the experiment.

#### 2.1.2. Information Intervention

To further understand how Beijing consumers’ preferences and willingness to pay (WTP) respond to new information, we designed three information treatments (in the form of messages disseminated with different contents). It has been documented that food consumers tend to have incorrect perceptions of the environmental impacts of food consumption [27], possess limited nutrition knowledge [42,43], and pay more attention to food safety [44,45]. Thus, we randomly provided the respondents with messages containing information on environmental, nutrition, and food safety issues related to the consumption of plant-based meat with a neutral tone (Table 1). The control group received no information treatment.

#### 2.1.3. Data Collection and Quality Control

A pilot survey involving 96 participants was conducted in early October 2021 to pre-test the quality of the survey questionnaire. The formal survey was administered in the second half of October 2021 using a stratified random sampling method on China’s largest online survey platform: Wen Juan Xing. Equivalent to Qualtrics, SurveyMonkey, or Cloud Research, this has a sample database covering over 6.0 million respondents whose personal information was confirmed, allowing for an authentic, diverse, and representative sample. Adults (aged 18 years and over) who are burger consumers located in Beijing were recruited for the survey. In the initial step, 687 respondents were randomly selected, and the final sample consisted of 526 respondents after quality control and manual checks to exclude incomplete and invalid questionnaires.

Before formally carrying out the choice experiment, we requested all participants to complete a survey on their socio-demographic characteristics and answer questions related to their perceptions of plant-based meat. To reduce the potential hypothetical bias [46], an “honest oath” was also included at the beginning of the survey. The respondents were asked to check the following statement before filling out the questionnaire: “I will provide honest answers throughout the experiment”.

### 2.2. Analytical Framework

#### 2.2.1. The Mixed-Logit Setup

Following the standard practice in the recent DCE literature, a mixed-logit model is adopted to analyze the data from the experiment. Compared with other discrete choice models such as multinominal logit, mixed-logit does not rely on the assumption of independence of irrelevant alternatives; thus, it can reflect a more realistic substitution pattern than standard logit models [47]. It also allows for autocorrelation among unobservable factors due to respondents’ repeated choices.

DCEs are developed based on the random-utility theory [48]. In a random-utility framework, consumer *n*’s utility ($U_{njt}$) derived from choosing burger *j* (*j* = 1,…, *J*) in scenario *t* (*t* = 1,…, *T*) can be specified as:

(1) $$U_{njt}=\beta^{\prime }x_{njt}+\varepsilon_{njt}$$

where $x_{njt}$ is the vector of observed attributes of burger *j* (*j* = 1,…, *J*) and $\varepsilon_{njt}$ represents unobservable components of $U_{njt}$. The probability that consumer *n* chooses burger *i* over *j* in scenario *t* is, then,

(2) $$P_{nit}=Prob\left(U_{nit}>U_{njt}\right),\forall j\neq i\;=Prob\left(\beta^{\prime }x_{nit}+\varepsilon_{nit}>\beta^{\prime }x_{njt}+\varepsilon_{njt}\right),\forall j\neq i\;=Prob\left(\varepsilon_{nit}-\varepsilon_{njt}>\beta^{\prime }x_{njt}-\beta^{\prime }x_{nit}\right),\forall j\neq i.$$

To proceed further, one needs to specify the functional form of $P_{nit}$. Following the common practice in the literature, we adopt a mixed-logit specification for $P_{nit}$, which allows the parameters (β) in the choice probability to follow a specific distribution rather than being fixed. More specifically, we parameterize the probability of consumer *n* choosing burger *i* in scenario *t*:

(3) $$P_{nit}=\int L_{nit}\left(\beta_{n}\right)f\left(\beta_{n}\right)d\left(\beta_{n}\right)$$

where

(4) $$L_{nit}\left(\beta_{n}\right)=\frac{exp\left(\beta_{n}^{\prime }x_{nit}\right)}{\sum_{j=1}^{J}exp\left(\beta_{n}^{\prime }x_{njt}\right)}$$

In this setup, $f\left(\beta_{n}\right)$ is a density function of the (random) parameters $\beta_{n}$, which may vary between consumers and follow a normal distribution; $L_{nit}$ represents the logit specification of the probability that consumer *n* chooses burger *i* in scenario *t* conditional on the realized value of $\beta_{n}$.

#### 2.2.2. Incorporating Information Effects

To further capture the effects of information treatments on consumers’ choices of burger alternatives, we specify the set of random coefficients $\beta_{n}$ to include the fixed mean value ($\bar{\beta }$) and three variable components, $Environment_{n}$, $Nutrition_{n}$, and $Food\_safety_{n}$, which are dummy variables indicating whether consumer *n* received the corresponding information treatment:

(5) $$\beta_{n}=\bar{\beta }+\delta_{1}Environment_{n}+\delta_{2}Nutrition_{n}+\delta_{3}Food\_safety_{n}+\sigma v_{n}$$

where $v_{n}$ stands for other unobserved factors assumed to follow a standard normal distribution, with σ being a scaling parameter. The probability of consumer *n* choosing burger *i* in scenario *t* is then the integral of standard logit probabilities over parameter densities:

(6) $$P_{nit}=\int \frac{exp\left(\beta_{n}^{\prime }x_{nit}\right)}{\sum_{j=1}^{J}exp\left(\beta_{n}^{\prime }x_{njt}\right)}f\left(v_{n}\right)d\left(v_{n}\right)$$

The corresponding log-likelihood function is given by:

(7) $$LL=\sum_{n=1}^{N}\sum_{j=1}^{J}d_{nit}lnP_{nit}$$

where $d_{nit}=1$ if consumer *n* chooses burger *i* and =0 otherwise. The parameters involved are estimated below by maximum likelihood estimation (MLE) in conjunction with 500 Halton draws.

Finally, to calculate consumers’ WTP for plant-based burgers and specific attributes, we divide the set of burger attributes into price ($price_{njt}$) and non-price attributes (${\overset{=}{x}}_{njt}$). The utility Function (1) can then be rewritten as:

(8) $$U_{njt}=\beta_{price}price_{njt}+{\overset{=}{\beta }}_{n}{}^{\prime }{\overset{=}{x}}_{njt}+\varepsilon_{njt}$$

where $\beta_{price}$ is the price parameter (assumed fixed), and ${\overset{=}{\beta }}_{n}$ is the set of coefficients on non-price attributes (${\overset{=}{x}}_{njt}$). Given this setup, the WTP for a non-price attribute of interest can be computed as:

(9) $$WTP_{non\_price\;attribute}=-\frac{{\overset{=}{\beta }}_{n}}{\beta_{price}}$$

## 3. Results

### 3.1. Distribution of Answers

As shown in Table 2, the largest group of respondents is between 26 and 30 years old, with a university degree, and a net household monthly income between RMB 10,000 and RMB 19,999 (≈ USD 1480–2960). While our respondents were younger and better-educated than the general population in Beijing, their age and education profiles are consistent with those of burger consumers in Beijing—the target population of our study [49]. Moreover, younger and better-educated consumers are most likely to be consumers of novel food products, including plant-based meat products [21]. The types of goods they consume, and their consumption patterns, have a typical exemplary effect and can predict social consumption trends [50]. Therefore, the findings obtained from our sample can provide support for the analysis of target consumer groups for plant-based meat products.

Table 3, Panel A, presents the sample respondents’ perceptions of plant-based meat using a 7-point Likert-type scale, with 1 indicating “totally disagree” and 7 indicating “totally agree”. The results suggest that the respondents’ overall attitude toward plant-based meat is on the positive side, as, on average, they “relatively agree” (>4) with positive statements (*a* and *e*) and “relatively disagree” (<4) with negative statements (*b*, *f* and *g*), even though they tend to believe that plant-based meat contains more additives than conventional meat (*d*).

Table 3, Panel B, reports the results of a test of consumers’ knowledge about plant-based meat. In the test, the respondents answered “True”, “False”, or “I don’t know” to a set of statements based on their own knowledge. Except for the first statement, the correct response rates are relatively low. In particular, only 12.36% of the participants responded correctly to the second statement, “Vegetarian chicken is a plant-based meat product”. Vegetarian chicken is a traditional soy-based food in China; although it is a product imitating meat, it has no meat-like flavor unless further seasoned or cooked, and thus not a plant-based meat product being examined in this study. This suggests that Beijing consumers are still confused about the difference between traditional Chinese artificial meat products and plant-based meat products. Although more than half of the participants provided correct responses to the other four questions, more than one-third responded with “I don’t know” to statements *c*, *d*, and *e*, and 22.62% to statement *a*. Only 3.42% of the participants answered all five questions correctly. These findings suggest that Beijing consumers’ knowledge about plant-based meat is still low.

### 3.2. Estimation Results of Mixed Logit Models

Table 4 reports the estimation results of mixed-logit models using the statistical package NLOGIT 6.0 with 500 Halton draws. Serving as the benchmark specification, Model 1 includes only the estimated random coefficients of burger attributes. Model 2 further allows consumers’ preferences to vary across information treatments by including interaction terms between burger attributes and information treatments―to avoid cluttering the table, only significant interaction terms are reported (the complete set of results is presented in Table A1). To put the impacts of information into perspective, Table 5 reports the mean WTP value for each attribute under different information treatments.

Model 1, the benchmark model, reveals three important findings. First, compared with conventional meat (the reference category for the “meat patty” attribute), consumers place significantly less value on plant-based alternative proteins (${\hat{\beta }}_{Plant\_based\_meat}$ = −0.30). For example, Table 5, column 1, suggests that the mean WTP for plant-based proteins is RMB -6.15 in the full sample. Second, meat patty flavor significantly influences consumers’ choices. Compared to chicken flavor (the reference category for the “flavor” attribute), beef flavor is more likely to be selected (${\hat{\beta }}_{Beef\_flavor}$ = 0.24, while pork flavor is less likely to be selected (${\hat{\beta }}_{Pork\_flavor}$ = −0.38). As shown in Table 5, column 1, consumers’ WTPs for pork and beef flavors are RMB -8.15 and RMB 5.27, respectively. Third, consumers preferred the lowest level of the patty’s sodium content (${\hat{\beta }}_{Sodium\_100\_mg}$ = 0.34) and burger’s energy (${\hat{\beta }}_{Cal\_250\_kcal}=0.29$) to their higher-level counterparts, reflecting their concerns about health when choosing burgers. Compared with other levels of the same attributes, respondents’ WTPs are highest for *Sodium_100_mg* and *Cal_250_kcal* (Table 5, column 1).

Model 2 reveals more instructive patterns regarding the role of information. Information treatments significantly impacted consumers’ choices, but the direction and magnitude of the impacts differed across treatments. A likelihood-ratio test rejected the null hypothesis that coefficients are equal across treatments at the 1% level (Table A2). The results suggest that information works through three attributes: *Meat patty* (plant-based meat), *Flavor* (beef flavor), and *Sodium content* (100, 250, and 400 mg). More specifically, the positive and statistically significant mean estimate of the “*Plant-based meat* × *Nutrition information*” interaction suggests that providing nutrition information induced more respondents to choose plant-based meat over animal meat. Echoing this effect, Table 5 indicates that the mean WTP for plant-based meat under the nutrition information treatment (column 3) is RMB 6.73 higher than that for the control group (column 2). This effect is reflected in the estimated confidence intervals of WTPs―note that the upper limit of the 95% confidence interval of WTP is positive for the nutrition information group, while those for other groups are all negative. By comparison, providing information on food safety (column 4) or the environment (column 5) had a somewhat smaller impact on the consumers’ preference for plant-based meat patties.

Nutrition information also had a positive effect on the consumers’ preference for sodium reduction (“*Sodium_550_mg*” is the reference group for the “*Sodium content*” attribute), as suggested by the positive and statistically significant coefficients of the “*Sodium_100_mg* × *Nutrition information*” and “*Sodium_400_mg* × *Nutrition information*” interactions (Table 4, model 2). Relative to the reference group, “*Sodium_550_mg*”, the respondents’ WTP for burgers with lower sodium content (100 mg and 400 mg) increased by more than 20% after receiving nutrition information (Table 5, column 3). Environmental information also helped enhance the consumers’ preference for sodium reduction. For example, as shown in Table 4, model 2, the estimated coefficient of the “*Sodium_400_mg* × *Environmental information*” interaction is positive and statistically significant. Correspondingly, Table 5, column 5, shows that the respondents’ WTP for *Sodium_400_mg* increased by 35.24% when provided with environmental information.

Interestingly, food safety information significantly *reduced* consumer preference for beef flavor. The coefficient of “*Beef flavor* × *Food safety information*” is negative and statistically significant (Table 4, model 2). Recall the above finding that consumers prefer beef flavor to chicken flavor without the information treatment. The negative coefficient of “*Beef flavor* × *Food safety information*” suggests that the provision of food safety information serves to equalize consumers’ preference for flavor.

## 4. Discussion

### 4.1. Findings and Implications

Our study found that Beijing consumers showed a low acceptance of plant-based meat products. This is not surprising given that the plant-based meat market is a “new animal in town”. Existing studies have pointed out that consumers are often hesitant to accept novel food or novel food technologies [51,52], thus having a low acceptance of novel alternative proteins [16,21,53,54]. Even in developed countries with vegetarian trends, the market shares of alternative proteins remain low compared to that of animal meat [55]. Focusing on China, Zhang et al. [56] observed that the overwhelming majority of consumers in Beijing, Qingdao, and Tai’an were unacquainted with artificial meat, and nearly 22% were opposed to artificial meat. Liu et al. [57] also concluded that 9.6% of Chinese consumers were “definitely unwilling” to try artificial meat, and 87.2% were willing to pay less for it compared to conventional meat. In line with the findings of previous studies, our study found that Beijing consumers had a low acceptance of and a negative WTP for plant-based meat.

However, information may promote Beijing consumers’ WTP for plant-based meat products. Consumers’ attitudes toward and preferences for plant-based meat can be altered by exogenously provided information. For example, consumers regard the lack of product information on the package, such as the origin of ingredients, as a disadvantage of meat substitutes [58]. Even though consumers are often unaware of the environmental and health problems associated with conventional meat consumption [59,60], claims related to improved human health can increase consumer acceptance of plant-based foods [28,61]. Claims of environmental benefits have also been shown to support the acceptance of plant-based protein alternatives such as seaweed [29], pulses [62], and lentils [63]. Information on the environment at least reduced the share of consumers unwilling to buy any plant-based products [21].

However, we found that while the nutrition information significantly impacted the consumers’ preferences for plant-based meat, which is in accord with the findings of Van Loo et al. [21] and Lusk and Briggeman [64], the effects of food safety information and environmental information were limited. A natural explanation is that consumers spontaneously make egoistic decisions, as altruistic decisions usually require deliberate control over oneself [65]. The contents of food safety information and environmental information are not as closely related to consumers’ personal interests as nutrition information; thus, they do not have a significant impact on consumers’ preferences for plant-based meat.

The findings of our study have important implications for the development of plant-based meat alternatives. The key challenge to developing a thriving plant-based meat market is how to achieve environmental benefits while satisfying consumers’ personal interests. To address this challenge, policy-makers may need to provide more information to deepen consumers’ understanding of plant-based meat products. Producers may focus on achieving nutritional sustainability in developing new plant-based meat products, such as providing products low in sodium and calorie contents. Marketing campaigns emphasizing the nutritional benefits may help induce consumers to choose plant-based meat products.

### 4.2. Limitations and Suggestions for Future Research

Before drawing conclusions, a note on the limitations of this study is in order. First, our survey over-sampled younger and better-educated consumers in Beijing. While younger and better-educated consumers are most likely to be consumers of novel food products, the findings based on such a sample may not be generalizable to all potential consumers in China. Future studies based on more representative samples are expected to be fruitful. Second, this is a stated preference study in nature. While such a design is necessary for examining the role of attributes that have not yet been incorporated into existing products on the market, the data collected in a hypothetical experiment may not reflect consumers’ actual behavior. For this reason, future research based on revealed preference data may help test the validity of our findings. Finally, participants in our experiment received only a one-time information intervention. Yet it is possible that consumers’ preferences for plant-based meat will change over repeated exposures. It would thus be helpful to conduct complementary studies involving repeated information interventions.

## 5. Conclusions

As part of the global strategy to address climate change and environmental degradation challenges, plant-based meat alternatives and related products have entered China’s food market. Since the market for plant-based meat in China is still in its infancy, a great many issues related to consumer preferences, food industry development, and relevant food policy remain to be explored. Based on a discrete choice experiment, this study has explored several key determinants of consumers’ preference for plant-based meat products in Beijing, China’s capital city. Our analysis found that Beijing consumers’ awareness of plant-based meat is rather limited, which may have led to a negative preference for plant-based meat among them. However, their preference (measured by their willingness to pay) for plant-based meat (as well as for attributes such as lower sodium and low calories) substantially increased after receiving information on the nutrition contents of plant-based meat products; in contrast, information on food safety and environmental issues related to plant-based meat consumption had little effect. Therefore, to promote plant-based meat consumption, at least in the context of Beijing, China, marketing campaigns may focus on providing information that is closely related to consumers’ personal interests rather than the “greater good”.

## Figures and Tables

**Table 1:** Contents of information treatments.

Treatments	Information Contents
Environmental information	Message 1: The livestock industry is a major contributor to global greenhouse gas emissions. Promoting a shift to a more sustainable global food system by reducing meat consumption and switching to plant-based diets will help reduce greenhouse gas emissions, save water and land resources, and promote environmental sustainability. Message 2: Currently, data on the benefits of plant-based products in reducing greenhouse gas emissions, lowering land and water consumption, and alleviating environmental burdens are limited.
Nutrition information	Message 1: Plant-based meat is rich in vitamins and can provide the same protein, minerals, and other nutrients as can those contained in animal meat. Plant-based meat is also rich in dietary fiber and overcomes many disadvantages of animal meat, such as excessive saturated fatty acids and high cholesterol. According to *Dietary Guidelines for Americans (2020–2025)*, the daily energy needs are 1600–2400 kcal for an adult female and 2000–3000 kcal for an adult male. Message 2: Compared with animal meat, plant-based meat lacks several essential nutrients, such as iron, zinc, and vitamin B12. Moreover, additional salt and other food additives need to be added for plant-based meat to mimic the flavor of animal meat. Consequently, the sodium content in plant-based meat tends to be higher than in animal meat. According to the *Dietary Guidelines for Americans (2020–2025)*, the daily sodium intake should not exceed 2300 mg, and should be further reduced for people under the age of 14.
Food safety information	Message 1: Consuming plant-based meat may avoid the risk of contracting diseases associated with animal meat consumption, such as mad cow disease and foot-and-mouth disease. Moreover, plant-based meat products are made without antibiotics and hormones. Message 2: There are relatively limited data and research on the safety of consuming plant-based meat products, such as microbial pollution, heavy metals residues, pesticide residues, etc. There is also a lack of data on the safety assessment of certain specific ingredients used in plant-based meat products.

Source: Author’s design.

**Table 2:** Socio-demographic characteristics of respondents.

Characteristics	Description	Percentage
Gender	Male	38.59
	Female	61.41
Age	20 or under	10.65
	21–25	15.59
	26–30	27.19
	31–35	20.72
	36–40	12.17
	41–45	6.08
	46–50	4.18
	51–55	0.95
	56–60	1.71
	61 or older	0.76
Education	Primary school	0.38
	Junior high school	1.90
	(Vocational) high school	6.46
	College/university	69.77
	Master’s degree or higher	21.48
Monthly income (RMB)	≤4999	9.89
	5000–9999	24.14
	10,000–19,999	36.31
	20,000–49,999	24.14
	50,000–99,999	3.42
	≥100,000	2.09

Note: N = 526. Source: author’s survey.

**Table 3:** Consumer perceptions of and knowledge about plant-based meat.

Statements	Mean (%)	SD
*A. Statements regarding plant-based meat*		
(a) It is safe to consume plant-based meat.	5.01	1.49
(b) Plant-based meat is toxic and/or carcinogenic.	2.54	1.54
(c) Plant-based meat has higher nutritional values.	3.67	1.67
(d) Plant-based meat contains more additives.	4.14	1.71
(e) Plant-based meat helps reduce CO_2_ emissions and mitigate climate change.	4.59	1.79
(f) The long-term impacts of plant-based meat consumption on the environment and human health are uncontrollable.	3.49	1.73
(g) I would rather avoid bad eating habits than choose plant-based meat foods.	3.33	1.92
*B. Knowledge about plant-based meat*	% Correct answers	
(a) At present, the raw materials of plant-based meat mainly include soybeans, wheat, and peas. (True)	75.29%	
(b) Vegetarian chicken is a plant-based meat product. (False) ^a^	12.36%	
(c) The research and development of plant-based meat-related products is prohibited in China. (False)	58.37%	
(d) Some brands of burgers have started using plant-based meat products. (True)	53.04%	
(e) Currently, there are plant-based meat dumplings, plant-based meat sausages, plant-based meatballs, and other products on the market. (True)	62.93%	

Source: author’s survey. Note: ^a.^ Vegetarian chicken is a traditional soy-based food in China. Although it is a product imitating meat, vegetarian chicken has no meat-like flavor unless further seasoned or cooked. Thus, it is not a plant-based meat product being examined in this study.

**Table 4:** Mixed-logit estimates.

	Model 1				Model 2			
	Mean of Parameter		SD of Parameter		Mean of Parameter		SD of Parameter	
*Attribute: meat patty*								
Plant-based meat	−0.303 ***	(0.093)	1.829 ***	(0.098)	−0.606 ***	(0.187)	1.832 ***	(0.098)
*Attribute: flavor*								
Pork flavor	−0.381 ***	(0.069)	0.909 ***	(0.084)	−0.261 *	(0.145)	0.927 ***	(0.085)
Beef flavor	0.237 ***	(0.072)	1.144 ***	(0.076)	0.422 ***	(0.146)	1.158 ***	(0.077)
(Reference category: Chicken flavor)								
*Attribute: sodium content*								
Sodium_100_mg	0.341 ***	(0.069)	0.844 ***	(0.073)	0.200	(0.142)	0.831 ***	(0.073)
Sodium_250_mg	0.209 ***	(0.066)	0.564 ***	(0.095)	0.148	(0.134)	0.579 ***	(0.094)
Sodium_400_mg	0.106	(0.067)	0.365 ***	(0.126)	−0.137	(0.139)	0.341 ***	(0.128)
(Reference category: Sodium_550_mg)								
*Attribute: energy*								
Cal_250_kcal	0.292 ***	(0.067)	0.738 ***	(0.069)	0.425 ***	(0.152)	0.744 ***	(0.069)
Cal_350_kcal	0.069	(0.059)	0.022	(0.174)	0.059	(0.136)	0.048	(0.169)
Cal_450_kcal	−0.032	(0.060)	0.003	(0.287)	0.025	(0.116)	0.030	(0.272)
(Reference category: Cal_550_kcal)								
*Interactions between attributes and information treatments (statistically significant terms only)*								
Plant-based meat × Nutrition information					0.574 **	(0.264)		
Beef flavor × Food safety information					−0.573 ***	(0.209)		
Sodium_100_mg × Nutrition information					0.488 **	(0.195)		
Sodium_400_mg × Environmental information					0.392 **	(0.199)		
Sodium_400_mg × Nutrition information					0.518 ***	(0.193)		
Price	−0.047 ***	(0.002)			−0.047 ***	(0.002)		
Alternative specific constant	−2.018 ***	(0.097)			−2.018 ***	(0.104)		
Log-likelihood	−5588.500				−5567.021			
AIC	11,217.0				11,228.0			
McFadden R^2^	0.175				0.178			
Number of observations	4208				4208			

Notes: Mixed-logit estimates for each of the treatment groups are reported in Table A2. Standard errors in parentheses. *** *p* < 0.01, ** *p* < 0.05, * *p* < 0.1.

**Table 5:** WTP estimates for different groups (unit: RMB).

	(1) Full Sample	(2) Control Group	(3) Nutrition Information Group	(4) Food Safety Information Group	(5) Environmental Information Group
*Attribute: meat patty*					
Plant-based Meat	−6.15 [−9.03, −3.26]	−8.84 [−14.71, −2.96]	−2.11 [−8.24, 4.03]	−6.42 [−12.21, −0.64]	−7.20 [−12.50, −1.90]
*Attribute: flavor*					
Pork flavor	−8.15 [−9.18, −7.11]	−7.93 [−10.05, −5.80]	−8.62 [−10.80, −6.45]	−8.50 [−10.41, −6.60]	−7.53 [−9.61, −5.45]
Beef flavor	5.27 [3.76, 6.77]	7.71 [4.66, 10.76]	4.19 [1.37, 7.02]	1.76 [−1.02, 4.53]	7.41 [4.19, 10.63]
*Attribute: sodium content*					
Sodium_100_mg	7.63 [6.64, 8.61]	7.32 [5.48, 9.16]	9.52 [7.40, 11.63]	7.86 [6.05, 9.66]	5.82 [3.74, 7.91]
Sodium_250_mg	4.39 [3.91, 4.86]	4.94 [4.05, 5.83]	4.01 [3.05, 4.96]	4.48 [3.42, 5.55]	4.11 [3.18, 5.04]
Sodium_400_mg	2.40 [2.17, 2.63]	2.08 [1.68, 2.48]	2.56 [2.00, 3.04]	2.13 [1.69, 2.57]	2.82 [2.32, 3.31]
*Attribute: energy*					
Cal_250_kcal	6.35 [5.53, 7.17]	6.66 [4.98, 8.34]	6.75 [5.06, 8.44]	5.71 [4.22, 7.20]	6.30 [4.57, 8.03]
Cal_350_kcal	1.47 [1.46, 1.47]	1.47 [1.45, 1.48]	1.47 [1.45, 1.48]	1.47 [1.46, 1.48]	1.46 [1.45, 1.48]
Cal_450_kcal	−0.67 [−0.67, −0.67]	−0.67 [−0.68, −0.67]	−0.67 [−0.67, −0.67]	−0.67 [−0.67, −0.67]	−0.67 [−0.68, −0.67]
N	526	131	131	132	132

Notes: Figures in brackets represent the 95% confidence intervals.

## Data Availability

Data are available from the corresponding author.

## Appendix A

**Table A1:** Mixed-logit model estimates.

	Model 1				Model 2			
	Mean of Parameter		SD of Parameter		Mean of Parameter		SD of Parameter	
*Attribute: meat patty*								
Plant-based Meat	−0.303 ***	(0.093)	1.829 ***	(0.098)	−0.606 ***	(0.187)	1.832 ***	(0.098)
*Attribute: flavor*								
Pork flavor	−0.381 ***	(0.069)	0.909 ***	(0.084)	−0.261 *	(0.145)	0.927 ***	(0.085)
Beef flavor	0.237 ***	(0.072)	1.144 ***	(0.076)	0.422 ***	(0.146)	1.158 ***	(0.077)
Reference category: chicken flavor								
*Attribute: flavor: sodium content*								
Sodium_100_mg	0.341 ***	(0.069)	0.844 ***	(0.073)	0.200	(0.142)	0.831 ***	(0.073)
Sodium_250_mg	0.209 ***	(0.066)	0.564 ***	(0.095)	0.148	(0.134)	0.579 ***	(0.094)
Sodium_400_mg	0.106	(0.067)	0.365 ***	(0.126)	−0.137	(0.139)	0.341 ***	(0.128)
(Reference category: Sodium_550_mg)								
*Attribute: energy*								
Cal_250_kcal	0.292 ***	(0.067)	0.738 ***	(0.069)	0.425 ***	(0.152)	0.744 ***	(0.069)
Cal_350_kcal	0.069	(0.059)	0.022	(0.174)	0.059	(0.136)	0.048	(0.169)
Cal_450_kcal	−0.032	(0.060)	0.003	(0.287)	0.025	(0.116)	0.030	(0.272)
Reference category: Cal_550_kcal								
*Interactions between attributes and information treatments*								
Plant-based Meat × Environmental information					0.312	(0.262)		
Plant-based Meat × Nutrition information					0.574 **	(0.264)		
Plant-based Meat × Food safety information					0.371	(0.263)		
Pork flavor × Environmental information					0.017	(0.201)		
Pork flavor × Nutrition information					−0.291	(0.193)		
Pork flavor × Food safety information					−0.225	(0.200)		
Beef flavor × Environmental information					0.075	(0.210)		
Beef flavor × Nutrition information					−0.365 *	(0.206)		
Beef flavor × Food safety information					−0.573 ***	(0.209)		
Sodium_100_mg × Environmental information					−0.058	(0.194)		
Sodium_100_mg × Nutrition information					0.488 **	(0.195)		
Sodium_100_mg × Food safety information					0.091	(0.194)		
Sodium_250_mg × Environmental information					0.066	(0.201)		
Sodium_250_mg × Nutrition information					0.155	(0.180)		
Sodium_250_mg × Food safety information					−0.072	(0.187)		
Sodium_400_mg × Environmental information					0.392 **	(0.199)		
Sodium_400_mg × Nutrition information					0.518 ***	(0.193)		
Sodium_400_mg × Food safety information					0.177	(0.194)		
Cal_250_kcal × Environmental information					−0.162	(0.210)		
Cal_250_kcal × Nutrition information					−0.026	(0.205)		
Cal_250_kcal × Food safety information					−0.147	(0.199)		
Cal_350_kcal × Environmental information					0.042	(0.198)		
Cal_350_kcal × Nutrition information					−0.155	(0.171)		
Cal_350_kcal × Food safety information					0.179	(0.180)		
Cal_450_kcal × Environmental information					0.067	(0.166)		
Cal_450_kcal × Nutrition information					−0.168	(0.163)		
Cal_450_kcal × Food safety information					−0.109	(0.172)		
Price	−0.047 ***	(0.002)			−0.047 ***	(0.002)		
Alternative Specific Constant	−2.018 ***	(0.097)			−2.018 ***	(0.104)		
Log likelihood	−5588.500				−5567.021			
AIC	11,217.0				11,228.0			
McFadden R^2^	0.175				0.178			
Number of observations	4208				4208			

Notes: Standard errors are presented in parentheses. *** *p* < 0.01, ** *p* < 0.05, * *p* < 0.1.

**Table A2:** Mixed-logit model estimates, by treatment groups.

		Control		Treatment 1-Environmental Information		Treatment 2-Nutrition Information		Treatment 3-Food Safety Information	
*Attribute: meat patty*									
Plant-based Meat	Mean	−0.539 **	(0.210)	−0.362 *	(0.185)	−0.078	(0.199)	−0.232	(0.186)
	SD	1.935 ***	(0.205)	1.534 ***	(0.168)	2.010 ***	(0.220)	1.814 ***	(0.196)
*Attribute: flavor*									
Pork flavor	Mean	−0.442 **	(0.174)	−0.255	(0.155)	−0.554 ***	(0.136)	−0.523 ***	(0.145)
	SD	1.172 ***	(0.181)	0.916 ***	(0.169)	0.969 ***	(0.162)	0.768 ***	(0.17)
Beef flavor	Mean	0.401 **	(0.156)	0.561 ***	(0.172)	0.068	(0.142)	−0.100	(0.151)
	SD	1.224 ***	(0.157)	1.353 ***	(0.168)	1.00 ***	(0.147)	1.071 ***	(0.154)
(Reference group: Chicken flavor)									
*Attribute: flavor: sodium content*									
Sodium_100_mg	Mean	0.285 *	(0.160)	0.237	(0.170)	0.460 **	(0.18)	0.267 *	(0.139)
	SD	0.745 ***	(0.158)	0.995 ***	(0.146)	1.011 ***	(0.158)	0.552 ***	(0.152)
Sodium_250_mg	Mean	0.224	(0.151)	0.201	(0.171)	0.284 **	(0.134)	0.014	(0.158)
	SD	0.394 *	(0.215)	0.720 ***	(0.185)	0.427 **	(0.188)	0.661 ***	(0.147)
Sodium_400_mg	Mean	−0.047	(0.151)	0.362 **	(0.165)	0.241	(0.170)	−0.070	(0.169)
	SD	0.046	(0.369)	0.595 ***	(0.195)	0.384	(0.235)	0.748 ***	(0.176)
(Reference group: Sodium_550_mg)									
*Attribute: energy*									
Cal_250_kcal	Mean	0.377 **	(0.165)	0.269	(0.169)	0.353 **	(0.153)	0.387 ***	(0.122)
	SD	0.913 ***	(0.155)	0.874 ***	(0.147)	0.761 ***	(0.154)	0.438 ***	(0.165)
Cal_350_kcal	Mean	0.106	(0.148)	0.121	(0.167)	−0.108	(0.126)	0.288 **	(0.123)
	SD	0.133	(0.293)	0.062	(0.159)	0.626 ***	(0.160)	0.100	(0.185)
Cal_450_kcal	Mean	−0.037	(0.121)	0.138	(0.152)	−0.161	(0.116)	−0.020	(0.143)
	SD	0.122	(0.311)	0.446 **	(0.225)	0.062	(0.205)	0.243	(0.293)
(Reference group: Cal_550_kcal)									
Price		−0.056 ***	(0.005)	−0.042 ***	(0.005)	−0.036 ***	(0.005)	−0.053 ***	(0.005)
Alternative Specific Constant		−2.337 ***	(0.193)	−1.744 ***	(0.285)	−1.876 ***	(0.204)	−2.052 ***	(0.200)
Log likelihood		−1330.611		−1378.758		−1420.812		−1411.905	
AIC		2701.2		2797.5		2881.6		2863.8	
McFadden *R*^2^		0.211		0.189		0.158		0.169	
Number of observations		1048		1056		1048		1056	

Notes: Standard errors are presented in parentheses. *** *p* < 0.01, ** *p* < 0.05, * *p* < 0.1.
